# Reader Comment Regarding “Cutaneous Immune-Related Adverse Events (irAEs) to Immune Checkpoint Inhibitors: A Dermatology Perspective on Management”

**DOI:** 10.1177/12034754211041610

**Published:** 2021-09-20

**Authors:** Maxwell Sauder, Marcus Butler

**Affiliations:** 1 10051 Princess Margaret Hospital Cancer Centre, Toronto, ON, Canada; 2 University of Toronto, ON, Canada

Over the past decade, immune checkpoint inhibitors (ICIs) have become a cornerstone in the management of cancer. Skin toxicities are the most common immune-related adverse event (irAE).^1^ As summarized by Muntyanu,^2^ cutaneous irAEs (cirAEs) are diseases that dermatologists diagnose and manage daily. The article highlights that we lack data demonstrating the most appropriate treatments of these toxicities that do not inhibit the anti-cancer effect.

Medical oncology guidelines generally recommend the use of systemic corticosteroids (sCSs) for grade 2 or 3 cirAEs.^3-5^ However, the mechanism of ICI anti-cancer effect is to activate the immune system, whereas sCSs are immunosuppressive. Earlier evidence suggested that sCSs do not prevent anti-cancer effects.^6,7^ However, these studies compare those experiencing irAEs to those who do not. Studies have demonstrated that patients experiencing irAEs have increased progression-free survival (PFS) and overall survival (OS).^8,9^ Further, high-dose sCSs for immune-related hypophysitis worsen PFS and OS.^10^ There is also direct evidence that use of sCS negatively impacts the ICI treatment effect.^11, 12^

In many cirAE presentations, there are alternatives to sCSs as summarized by Muntyanu. Therefore, management of cirAEs without sCS should be considered, especially for disorders with suitable alternatives.

References available online via Supplemental Material.

## Supplemental Material

Online supplementary file 1 - Supplemental material for Reader Comment Regarding “Cutaneous Immune-Related Adverse Events (irAEs) to Immune Checkpoint Inhibitors: A Dermatology Perspective on Management” Click here for additional data file. Supplemental material, Online supplementary file 1, for Reader Comment Regarding “Cutaneous Immune-Related Adverse Events (irAEs) to Immune Checkpoint Inhibitors: A Dermatology Perspective on Management” by Maxwell Sauder and Marcus Butler in Journal of Cutaneous Medicine and Surgery

