# Supplementary material for: Reader Comment Regarding “Cutaneous Immune-Related Adverse Events (irAEs) to Immune Checkpoint Inhibitors: A Dermatology Perspective on Management”
Source: J Cutan Med Surg. 2021 Sep 20;26(1):105. doi: 10.1177/12034754211041610 (PMC8750150; doi:10.1177/12034754211041610)
Supplement: Online supplementary file 1 - Supplemental material for Reader Comment Regarding “Cutaneous Immune-Related Adverse Events (irAEs) to Immune Checkpoint Inhibitors: A Dermatology Perspective on Management” [file sj-docx-1-cms-10.1177_12034754211041610.docx]

**Supplemental References**

**Reader Comment Regarding “Cutaneous Immune-Related Adverse Events (irAEs) to Immune Checkpoint Inhibitors: A Dermatology Perspective on Management”**

Maxwell Sauder, MD and Marcus Butler, MD

1. Puzanov I, Diab A, Abdallah K, Bingham CO 3rd, Brogdon C, Dadu R, Hamad L, Kim S, Lacouture ME, LeBoeuf NR, Lenihan D, Onofrei C, Shannon V, Sharma R, Silk AW, Skondra D, Suarez-Almazor ME, Wang Y, Wiley K, Kaufman HL, Ernstoff MS; Society for Immunotherapy of Cancer Toxicity Management Working Group. Managing toxicities associated with immune checkpoint inhibitors: consensus recommendations from the Society for Immunotherapy of Cancer (SITC) Toxicity Management Working Group. J Immunother Cancer. 2017 Nov 21;5(1):95. doi: 10.1186/s40425-017-0300-z. PMID: 29162153; PMCID: PMC5697162.
2. Muntyanu A, Netchiporouk E, Gerstein W, Gniadecki R, Litvinov IV. Cutaneous Immune-Related Adverse Events (irAEs) to Immune Checkpoint Inhibitors: A Dermatology Perspective on Management [Formula: see text]. J Cutan Med Surg. 2021 Jan-Feb;25(1):59-76. doi: 10.1177/1203475420943260. Epub 2020 Aug 3. PMID: 32746624.
3. Thompson JA, Schneider BJ, Brahmer J, Andrews S, Armand P, Bhatia S, Budde LE, Costa L, Davies M, Dunnington D, Ernstoff MS, Frigault M, Kaffenberger BH, Lunning M, McGettigan S, McPherson J, Mohindra NA, Naidoo J, Olszanski AJ, Oluwole O, Patel SP, Pennell N, Reddy S, Ryder M, Santomasso B, Shofer S, Sosman JA, Wang Y, Weight RM, Johnson-Chilla A, Zuccarino-Catania G, Engh A. NCCN Guidelines Insights: Management of Immunotherapy-Related Toxicities, Version 1.2020. J Natl Compr Canc Netw. 2020 Mar;18(3):230-241. doi: 10.6004/jnccn.2020.0012. PMID: 32135517.
4. Brahmer JR, Lacchetti C, Schneider BJ, Atkins MB, Brassil KJ, Caterino JM, Chau I, Ernstoff MS, Gardner JM, Ginex P, Hallmeyer S, Holter Chakrabarty J, Leighl NB, Mammen JS, McDermott DF, Naing A, Nastoupil LJ, Phillips T, Porter LD, Puzanov I, Reichner CA, Santomasso BD, Seigel C, Spira A, Suarez-Almazor ME, Wang Y, Weber JS, Wolchok JD, Thompson JA; National Comprehensive Cancer Network. Management of Immune-Related Adverse Events in Patients Treated With Immune Checkpoint Inhibitor Therapy: American Society of Clinical Oncology Clinical Practice Guideline. J Clin Oncol. 2018 Jun 10;36(17):1714-1768. doi: 10.1200/JCO.2017.77.6385. Epub 2018 Feb 14. PMID: 29442540; PMCID: PMC6481621.
5. Haanen JBAG, Carbonnel F, Robert C, Kerr KM, Peters S, Larkin J, Jordan K; ESMO Guidelines Committee. Management of toxicities from immunotherapy: ESMO Clinical Practice Guidelines for diagnosis, treatment and follow-up. Ann Oncol. 2017 Jul 1;28(suppl_4):iv119-iv142. doi: 10.1093/annonc/mdx225. Erratum in: Ann Oncol. 2018 Oct 1;29(Suppl 4):iv264-iv266. Erratum in: Ann Oncol. 2018 Oct;29 Suppl 4:iv264-iv266. PMID: 28881921.
6. Weber JS, Hodi FS, Wolchok JD, Topalian SL, Schadendorf D, Larkin J, Sznol M, Long GV, Li H, Waxman IM, Jiang J, Robert C. Safety Profile of Nivolumab Monotherapy: A Pooled Analysis of Patients With Advanced Melanoma. J Clin Oncol. 2017 Mar;35(7):785-792. doi: 10.1200/JCO.2015.66.1389. Epub 2016 Nov 14. PMID: 28068177.
7. Petrelli F, Signorelli D, Ghidini M, Ghidini A, Pizzutilo EG, Ruggieri L, Cabiddu M, Borgonovo K, Dognini G, Brighenti M, De Toma A, Rijavec E, Garassino MC, Grossi F, Tomasello G. Association of Steroids use with Survival in Patients Treated with Immune Checkpoint Inhibitors: A Systematic Review and Meta-Analysis. Cancers (Basel). 2020 Feb 27;12(3):546. doi: 10.3390/cancers12030546. PMID: 32120803; PMCID: PMC7139305.
8. Teulings HE, Limpens J, Jansen SN, Zwinderman AH, Reitsma JB, Spuls PI, Luiten RM. Vitiligo-like depigmentation in patients with stage III-IV melanoma receiving immunotherapy and its association with survival: a systematic review and meta-analysis. J Clin Oncol. 2015 Mar 1;33(7):773-81. doi: 10.1200/JCO.2014.57.4756. Epub 2015 Jan 20. PMID: 25605840.
9. Ricciuti B, Genova C, De Giglio A, Bassanelli M, Dal Bello MG, Metro G, Brambilla M, Baglivo S, Grossi F, Chiari R. Impact of immune-related adverse events on survival in patients with advanced non-small cell lung cancer treated with nivolumab: long-term outcomes from a multi-institutional analysis. J Cancer Res Clin Oncol. 2019 Feb;145(2):479-485. doi: 10.1007/s00432-018-2805-3. Epub 2018 Dec 1. PMID: 30506406.
10. Faje AT, Lawrence D, Flaherty K, Freedman C, Fadden R, Rubin K, Cohen J, Sullivan RJ. High-dose glucocorticoids for the treatment of ipilimumab-induced hypophysitis is associated with reduced survival in patients with melanoma. Cancer. 2018 Sep 15;124(18):3706-3714. doi: 10.1002/cncr.31629. Epub 2018 Jul 5. PMID: 29975414.
11. Eggermont AMM, Kicinski M, Blank CU, Mandala M, Long GV, Atkinson V, Dalle S, Haydon A, Khattak A, Carlino MS, Sandhu S, Larkin J, Puig S, Ascierto PA, Rutkowski P, Schadendorf D, Koornstra R, Hernandez-Aya L, Di Giacomo AM, van den Eertwegh AJM, Grob JJ, Gutzmer R, Jamal R, Lorigan PC, Krepler C, Ibrahim N, Marreaud S, van Akkooi A, Robert C, Suciu S. Association Between Immune-Related Adverse Events and Recurrence-Free Survival Among Patients With Stage III Melanoma Randomized to Receive Pembrolizumab or Placebo: A Secondary Analysis of a Randomized Clinical Trial. JAMA Oncol. 2020 Apr 1;6(4):519-527. doi: 10.1001/jamaoncol.2019.5570. PMID: 31895407; PMCID: PMC6990933.
12. Bai X, Hu J, Betof Warner A, et al. Early use of high-dose-glucocorticoid for the management of irAE is associated with poorer survival in patients with advanced melanoma treated with anti-PD-1 monotherapy. *Clin Cancer Res*. 2021 Aug 10:clincanres.1283.2021. doi: 10.1158/1078-0432.CCR-21-1283. Epub ahead of print. PMID: 34376536.
